# Evolutionary Strategies for Heavy Metal Resistance: Genomic Plasticity in *Pseudomonas* Versus Stability in *Aeromonas* and *Bacillus*

**DOI:** 10.3390/biology15100751

**Published:** 2026-05-09

**Authors:** Di Peng, Tao Huang, Wei Kang

**Affiliations:** 1 East China Sea Fisheries Research Institute, Chinese Academy of Fishery Sciences, Shanghai 200090, China; pengdi@ecsf.ac.cn (D.P.); sjtu2409180@sjtu.edu.cn (T.H.); 2 Key Laboratory of Environmental Health Impact Assessment of Emerging Contaminants, Ministry of Ecology and Environment, School of Environmental Science and Engineering, Shanghai Jiao Tong University, Shanghai 200240, China

**Keywords:** *Pseudomonas*, *Bacillus*, *Aeromonas*, heavy metals, evolution, pan-genome

## Abstract

Heavy metal resistance represents a critical microbial trait shaped by lineage-specific evolutionary pressures, yet its genomic foundations and diversification across major bacterial taxa remain poorly resolved. This study presented a comparative pangenomic analysis of *Aeromonas* (n = 32), *Bacillus* (n = 123), and *Pseudomonas* (n = 350)—three phylogenetically and ecologically distinct genera frequently enriched in metal-contaminated environments and exhibiting notable differences in resistance architectures. All three genera exhibited open pangenomes, with fitted expansion indices of 0.003 (*Aeromonas*), 0.03 (*Bacillus*), and 0.04 (*Pseudomonas*), each showing strong model fit (R^2^ > 0.98). *Pseudomonas* harbored a significantly greater number of resistance genes, with copper and zinc resistance genes exceeding 25 per strain in some cases. Most heavy metal resistance genes across the three genera were subject to purifying selection (dN/dS < 1), and no significant expansion or contraction of these gene families was observed (*p* > 0.05). The presence of these genera and their lineage-specific resistance determinants may serve as bioindicators of heavy metal exposure, offering valuable references for assessing contamination levels through environmental metagenomics.

## Simple Summary

Heavy metal pollution severely threatens our ecosystems and health. To understand natural defense mechanisms, this study explores how three common bacteria—*Pseudomonas*, *Bacillus*, and *Aeromonas*—adapt their genetic material to resist metal toxicity. By comparing their DNA, we found distinct survival strategies. *Pseudomonas* has the most flexible and abundant resistance genes, especially against copper and zinc. *Bacillus* adapts by altering its nutritional metabolism, while *Aeromonas* relies on a highly stable genetic foundation. We conclude that these bacteria use diverse evolutionary strategies to survive metal stress. These findings are valuable to society because these bacteria and their unique resistance traits can serve as biological indicators, helping us easily detect and measure heavy metal pollution levels in natural environments.

## 1. Introduction

Heavy metal pollution threatens ecosystems and human health due to the toxicity and persistence of elements such as cadmium (Cd), lead (Pb), arsenic (As), and mercury (Hg) [1,2]. These pollutants exert strong and sustained selective pressure on microbial communities, driving the evolution of diverse genetic strategies for metal tolerance, including efflux systems, metal sequestration, and enzymatic detoxification [3,4,5]. Unlike conventional remediation technologies, which are often hindered by high costs, operational complexity, and secondary pollution risks [6], microbial resistance mechanisms arise naturally and are encoded in well-conserved genomic frameworks. Among the microbes adapted to metal-contaminated environments, the genera *Pseudomonas*, *Bacillus*, and *Aeromonas* are frequently detected in metal-rich soils and aquatic systems, reflecting their pronounced resistance capacities and genomic plasticity [7]. These genera encode structured resistance systems—for example, CzcCBA efflux pumps and P-type ATPases in *Pseudomonas* [8], cadA-type genes in *Bacillus*, and *copA*/*czcA* in *Aeromonas* [9]—conferring resistance to Cu, Zn, and Cd. Their presence in metagenomic datasets may serve as bioindicators of heavy metal exposure, providing a molecular proxy for contamination levels.

Traditional 16S rRNA-based taxonomy lacks sufficient resolution in several of our focal genera—for example, members of the *Pseudomonas* putida/fluorescens groups often cluster together by 16S and require housekeeping-gene or whole-genome markers (e.g., *rpoD*, *gyrB*) for reliable identification [10]. Similarly, the *Bacillus* cereus sensu lato group is nearly indistinguishable by 16S [11], and *Aeromonas* is often resolved only to the genus level [12]. Such taxonomic ambiguity can blur lineage–trait links for heavy-metal resistance—masking clade-specific distributions of efflux pumps, redox systems, and siderophore/non-ribosomal peptide (NRP) pathways—and may limit the interpretation of ecological adaptation and resistance potential [13]. In this context, high-resolution phylogenomics and pangenome frameworks are essential not only for resolving species boundaries but also for linking resistance gene repertoires with their taxonomic carriers. Establishing such lineage-resolved associations is critical if microbial taxa and their resistance determinants are to be used as bioindicators of metal exposure. Moreover, as metagenomic surveys increasingly detect these resistance signatures in environmental samples, the integration of genomic context allows them to be interpreted as molecular proxies for contamination gradients [14,15].

The heavy metal resistance of *Pseudomonas*, *Bacillus*, and *Aeromonas* is primarily conferred by their genomes, which are enriched with diverse metal resistance genes [7]. Although pangenome frameworks have been applied in several bacterial taxa, it remains unclear whether *Pseudomonas*, *Bacillus*, and *Aeromonas* exhibit comparable levels of genome openness, and whether such differences align with their ecological adaptation to heavy-metal stress. Such resistance genes facilitate ecological adaptation and persistence in heavy metal-impacted habitats, and their lineage-specific distribution patterns may also serve as potential bioindicators of metal exposure, linking genomic signatures to environmental contamination signals detectable in metagenomic datasets [14,15]. Despite the availability of extensive whole-genome sequences for these genera in public databases, pangenomic investigations remain relatively limited. By contrast, extensive studies have been conducted in other bacteria, such as *Pseudomonas aeruginosa* [16], *Bacillus* species [17], *Flavobacterium* [18], and *Bacteroides* [19], revealing strain-level diversity, gene family expansion, and ecological adaptation. However, similar analyses focusing on heavy metal resistance remain scarce for *Aeromonas*, *Bacillus*, and *Pseudomonas*. Pangenome analyses are essential for revealing genetic diversity within and across species, offering insights into their adaptive mechanisms and evolutionary trajectories under diverse environmental pressures [18]. Notably, many resistance mechanisms overlap with secondary-metabolite biosynthetic pathways, such as non-ribosomal peptides and siderophores. However, whether this co-occurrence reflects a systematic, lineage-dependent trend across the three genera has not yet been established. Comparative genome and pangenome analyses have identified key genes associated with heavy metal resistance and clarified the mechanisms underlying their dissemination and retention in microbial populations [20]. Functional diversification is often accompanied by distinct evolutionary pressures. It remains uncertain whether heavy-metal resistance genes in these genera are largely constrained by purifying selection or whether adaptive signals emerge in specific lineages or metal-related functions. These uncertainties highlight the importance of establishing genomic baselines that enable resistance gene profiles to be interpreted not only in evolutionary terms but also as molecular proxies for assessing heavy metal stress in natural environments.

Understanding how metal stress drives genomic adaptation—such as the expansion or contraction of defense- and metabolism-related gene families—is crucial for elucidating the evolutionary basis of bacterial metal resistance. Recent genome-scale and pangenomic analyses have revealed species-specific mechanisms, including efflux systems, redox enzymes, and mobile resistance elements, but these efforts have largely focused on single taxa or metal types [16,18,21,22]. Here, we extend this framework by systematically comparing *Aeromonas*, *Bacillus*, and *Pseudomonas*, integrating pangenomic and evolutionary analyses to uncover genus-specific adaptive strategies shaped by distinct metal exposure histories. Building on this foundation, the present study aims to conduct a comprehensive comparative analysis of the pangenomes and core genomes of 350 *Pseudomonas*, 123 *Bacillus*, and 32 *Aeromonas* strains. To the best of our knowledge, this is the first systematic pangenomic comparison of *Pseudomonas*, *Bacillus*, and *Aeromonas* in the context of heavy metal resistance. By integrating phylogenetic reconstruction, gene family clustering, resistance gene annotation, and evolutionary rate analyses, this study reveals genus-specific patterns of genome dynamics and resistance gene evolution under metal stress, thereby providing both ecological insights and a genomic reference framework for interpreting resistance determinants as bioindicators in environmental metagenomes.

## 2. Materials and Methods

### 2.1. Gene and Protein Sequence Acquisition and Selection

Genomic and protein sequences of *Aeromonas*, *Bacillus* and *Pseudomonas* were retrieved from the NCBI database using the ncbi-genome-download (https://github.com/kblin/ncbi-genome-download/, accessed on 26 April 2026, v0.3.3) tool. Initially, representative reference genomes were selected from the downloaded dataset for each species. Genome quality was then assessed using CheckM2 (v1.1.0) [23] to ensure data integrity. Specifically, genomes with completeness values greater than 90% and contamination rates below 5% were retained. Following this stringent selection process, high-quality genomic and protein sequences from 32 *Aeromonas* strains, 123 *Bacillus* strains, and 350 *Pseudomonas* strains were obtained for further analysis.

### 2.2. Phylogenetic Tree Construction

To construct a phylogenetic tree with sufficient resolution and taxonomic representation, 120 widely conserved single-copy bacterial proteins were extracted from all genomes. This was accomplished using the classify_wf module of GTDB-Tk (v2.4.0) with the reference database set to R220 [24]. Tree inference was performed using IQ-TREE2 (v2.4.0) [25] with the parameters -m MFP -nt 60 -bb 1000 -redo -mredo. The resulting phylogenetic tree was visualized using iTOL (v7) [26], following the generation of the necessary configuration file via the itol.toolkit [27].

### 2.3. Homologous Gene Extraction

Homologous gene families were identified using OrthoFinder (v2.5.5) [28], employing the following settings: -S diamond -M msa -A mafft -T fasttree -t 16. The genes were then classified into core, accessory, and unique categories. Core genes were defined as those present in all genomes, accessory genes as those present in at least two but not all genomes, and unique genes as those found exclusively in individual strains. To model the gene content dynamics of the pan-genome and core genome, PanGP (v1.0.160) [29] was used based on the OrthoFinder output. The simulation was performed using the complete random algorithm, with a sample size of 1000 and 30 repetitions.

### 2.4. Functional Annotation

Functional annotations were carried out using EggNOG-mapper [30], which assigned functional categories to all pan-genome genes based on the COG database. Enrichment significance was evaluated using Fisher’s exact test. Additionally, to identify metal-related genes, BLASTp (v2.2.1) [31] searches were performed using the BacMet2_EXP_database [14] as the reference. The parameters were set as: --outfmt 6 --evalue 1 × 10^−5^ -p 40 --max-target-seqs 1 --min-score 60 --id 40 --query-cover 20 [32].

### 2.5. Gene Evolution Analysis

To assess selective pressure on gene families, the codeML module of PAML [33] was used to estimate the ratio of nonsynonymous to synonymous substitution rates (dN/dS) for both core and accessory genes. Furthermore, gene family expansions and contractions were evaluated using CAFE5 [34], with a significance threshold set at *p* < 0.05.

### 2.6. Computational Environment, Visualization, and Statistical Analysis

All computational analyses were performed on a high-performance workstation equipped with an AMD EPYC 7763 CPU (64 cores, 128 threads) and 512 GB of RAM. Data processing and figure generation were carried out using Python 3.10 with the libraries Matplotlib (v3.10.1) and Seaborn (v2.5.5), as well as OriginLab 2023 for advanced graphical plotting. All statistical analyses were performed using SPSS Statistics v27.0 and Python 3.10 (libraries: SciPy, statsmodels). Comparisons of gene abundance across genera were conducted using one-way ANOVA, followed by Tukey’s post hoc test to identify pairwise differences. For categorical data, such as the presence/absence of gene families or secondary metabolite clusters, Fisher’s exact test was applied. Principal Component Analysis (PCA) and pan-genome accumulation curves were statistically assessed by comparing variance between groups and goodness-of-fit (R^2^) values. Reported *p*-values correspond to the specific test applied for each analysis, with significance thresholds set at *p* < 0.05, *p* < 0.01, and *p* < 0.001. Figures and Supplementary Materials indicate which statistical tests were used for each dataset, ensuring reproducibility and transparency.

## 3. Results and Discussion

### 3.1. Genome Statistics and Phylogenetic Analysis

**Figure 1:**
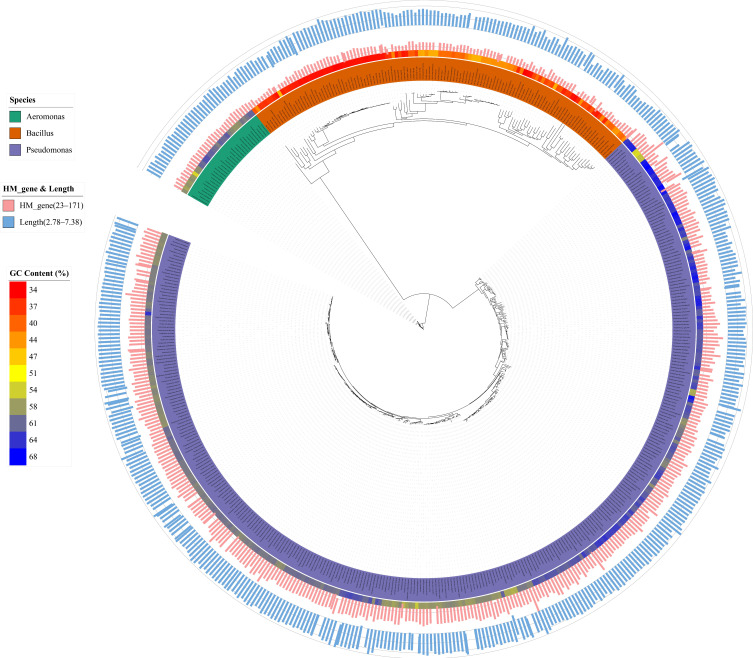
Phylogenetic tree of 350 *Pseudomonas*, 123 *Bacillus*, and 32 *Aeromonas* strains based on 120 single-copy conserved genes extracted from whole genomes using GTDB-Tk. Branches are colored by genus, and major clades are highlighted. Gene length, number of metal resistance genes per strain, and GC content were calculated from the genome assemblies. The tree illustrates clear genus-level clustering, reflecting evolutionary divergence and potential use as taxonomic and genomic indicators of metal exposure.

The phylogenetic analysis (Figure 1) demonstrated that *Aeromonas*, *Bacillus*, and *Pseudomonas* formed three distinct clades. The genomes of *Aeromonas* were tightly clustered, indicating a high degree of genetic similarity within the genus. In contrast, *Bacillus* displayed a more dispersed distribution, reflecting greater genomic diversity, while *Pseudomonas* exhibited the broadest distribution, suggesting extensive adaptability to diverse environments and a complex evolutionary history. Lineage-specific clustering illustrates evolutionary divergence and provides a basis for using these taxa as bioindicators, as their occurrence patterns reflect selective pressures from heavy metal exposure.

**Figure 2:**
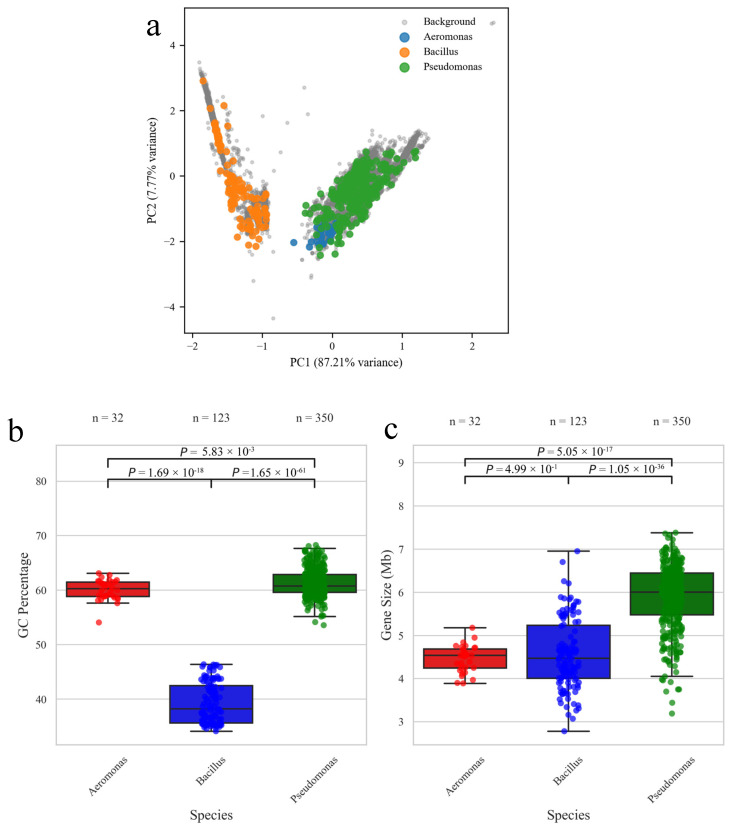
Comparative genomic statistics of *Pseudomonas*, *Bacillus*, and *Aeromonas*. (**a**) PCA of genome features, including genome size, GC content, and accessory gene counts, showing distinct clustering by genus. (**b**) GC content distribution of genes across the three genera: *Pseudomonas* exhibits the highest GC content, followed by *Aeromonas* and *Bacillus*. (**c**) Genome size distribution: *Aeromonas* shows a relatively small and stable genome (~4.5 Mb), *Bacillus* exhibits a wider range (4–6.5 Mb), and *Pseudomonas* harbors the largest genomes (6–7 Mb), indicating genomic complexity and potential adaptability. Data derived from pan-genome analysis of all included strains.

Significant differences in GC content were also observed among the three genera. Both *Aeromonas* and *Pseudomonas* showed relatively high GC content (approximately 60%), whereas *Bacillus* had a considerably lower value (around 38%). The GC content analysis (Figure 2b) confirmed that *Pseudomonas* possessed the highest GC content, followed by *Aeromonas* and then *Bacillus*. These inter-genus differences were likely to reflect underlying variations in genomic organization and functional capacity [35,36,37]. High GC content may further contribute to the stability of essential genetic elements under heavy metal stress, as metal exposure was often associated with increased DNA damage [38]. From a monitoring perspective, the consistent distinction in GC profiles across these genera means that their enrichment in metagenomic datasets can serve not only as taxonomic markers but also as indirect indicators of stress-adapted microbial communities in contaminated habitats.

In terms of genome size (Figure 2c), *Aeromonas* exhibited a relatively small and stable genome, with an average size of approximately 4.5 Mb [36]. In comparison, *Pseudomonas* harbored larger genomes, typically ranging from 6 to 7 Mb, indicative of greater genetic content and complexity. *Bacillus* showed a wider variation in genome size, ranging from 4 Mb to 6.5 Mb, suggesting higher genomic diversity and adaptability. Notably, *Pseudomonas* tended to contain a larger repertoire of heavy metal resistance genes, which may reflect its environmental exposure to heavy metal contamination [7]. Thus, the larger genome size of *Pseudomonas*, coupled with its higher proportion of accessory genome content, may contribute to its extensive repertoire of metal resistance genes [39,40]. By contrast, *Bacillus* and *Aeromonas* also carried such genes but in more consistent and relatively smaller numbers, respectively. This large resistance repertoire also provides a measurable genomic signature: detecting elevated frequencies of such genes in metagenomic surveys could act as a proxy for high metal load in environmental samples.

Furthermore, PCA (Figure 2a) provided additional insights into genomic differences among the three genera. The positions of *Aeromonas*, *Bacillus* and *Pseudomonas* were clearly separated in the PCA plot, consistent with the phylogenetic tree results. This separation suggests the relative independence of their genomic features. The species were evenly distributed against the background genome, confirming that the selected strains were representative of the genomic characteristics of their respective genera. Importantly, *Pseudomonas* genomes occupied a distinct position, further highlighting their unique genomic architecture and extensive diversity. These findings were consistent with the GC content analysis and may also be explained by the taxonomic distinction that *Aeromonas* and *Pseudomonas* are Gram-negative bacteria, whereas *Bacillus* was Gram-positive [41]. In practical terms, such distinct clustering implies that *Aeromonas*, *Bacillus*, and *Pseudomonas* can be tracked in metagenomic datasets as taxonomic and genomic sentinels, whose abundance and resistance profiles may reveal the extent and nature of local heavy metal pollution.

### 3.2. Metal Resistance and Secondary Metabolism Gene Clusters

**Figure 3:**
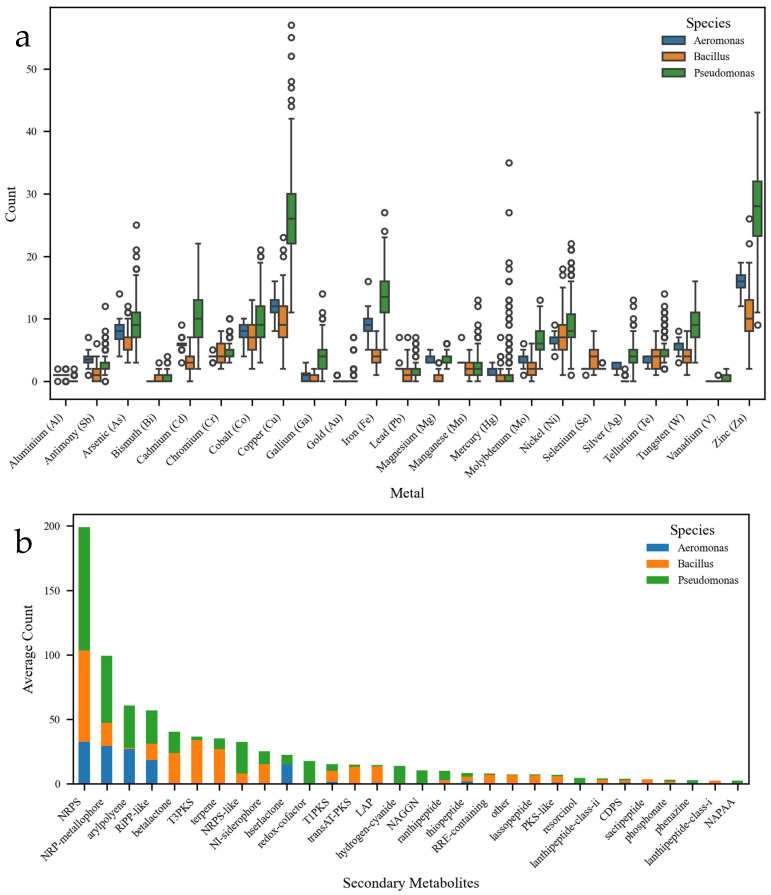
Comparative analysis of functional gene content related to metal resistance and secondary metabolism. (**a**) Number of heavy metal resistance genes across *Pseudomonas*, *Bacillus*, and *Aeromonas* strains. Median values and ranges indicate that *Pseudomonas* harbors the largest repertoire, with particular enrichment in Cu and Zn resistance. (**b**) Secondary metabolite gene clusters in the three genera, including non-ribosomal peptide (NRP), RiPP-like, arylpolyene, and NRP-metallophore clusters. *Pseudomonas* exhibits the highest diversity and abundance, followed by *Bacillus*, while *Aeromonas* shows the fewest clusters, reflecting differences in biosynthetic capacity and potential adaptation to heavy metal stress. Data derived from pan-genome analysis of 350, 123, and 32 strains, respectively.

The number of heavy metal resistance genes was significantly higher in *Pseudomonas* compared with *Aeromonas* and *Bacillus* (Figure 3a). For most heavy metals, the median values and distribution ranges of resistance genes in *Pseudomonas* exceeded those observed in the other two genera. Although *Bacillus* possessed slightly more resistance genes than *Aeromonas*, the numbers were still considerably lower than those in *Pseudomonas*. This stratification in resistance gene abundance suggests that genus-specific profiles could be used as bioindicators in metagenomic surveys [42]. Overall, *Pseudomonas* displayed a clear advantage in adapting to environments contaminated with diverse heavy metals, likely due to its extensive repertoire of resistance genes. Importantly, the interspecies differences were most evident for particular metals, with Cu and Zn showing the highest gene counts. These findings were consistent with previous studies demonstrating that *Pseudomonas* species had evolved resistance to a broad spectrum of heavy metals, facilitated by genetic mechanisms such as efflux pumps, metal chelation, and enzymatic detoxification [21,22]. In contrast, *Aeromonas* and *Bacillus* [43] appeared to harbor more specialized resistance genes, which may constrain their adaptability in environments heavily contaminated with multiple heavy metals [43,44]. Such differences provide a basis for using resistance gene repertoires as molecular proxies of contamination levels.

Comparative analysis of secondary metabolism-related gene clusters revealed striking interspecies differences among the three genera (Figure 3b). *Pseudomonas* contained a higher number of clusters for most secondary metabolites, highlighting its greater richness and diversity in secondary metabolite biosynthesis [45]. *Bacillus* harbored more clusters for certain metabolite types, while *Aeromonas* had the fewest, reflecting its relatively limited biosynthetic capacity. Of particular note, *Pseudomonas* possessed an exceptionally high number of NRP gene clusters, with an average approaching 200, underscoring its substantial advantage in NRP biosynthesis. Because many NRPs and metallophores bind metal ions [46], their co-occurrence with resistance genes may serve as additional genomic signals of heavy metal pressure in metagenomic datasets. This capacity likely provides *Pseudomonas* with a competitive edge in colonizing diverse ecological niches. Moreover, *Pseudomonas* also showed a remarkable enrichment of NRP-metallophore gene clusters, with the highest diversity in metal ion binding [46]. This observation is consistent with the results in Figure 3a and further emphasizes the tight link between metal resistance and secondary metabolite biosynthesis in this genus. In addition to NRP and NRP-metallophore clusters, RiPP-like and arylpolyene clusters ranked in the third or fourth place in abundance, and both *Pseudomonas* and *Aeromonas* carried relatively high levels of these, compared with *Bacillus*. Such lineage-specific associations strengthen their potential as indicators when interpreting resistance signatures from metagenomic data.

### 3.3. Pan-Genome Construction and Analysis

**Figure 4:**
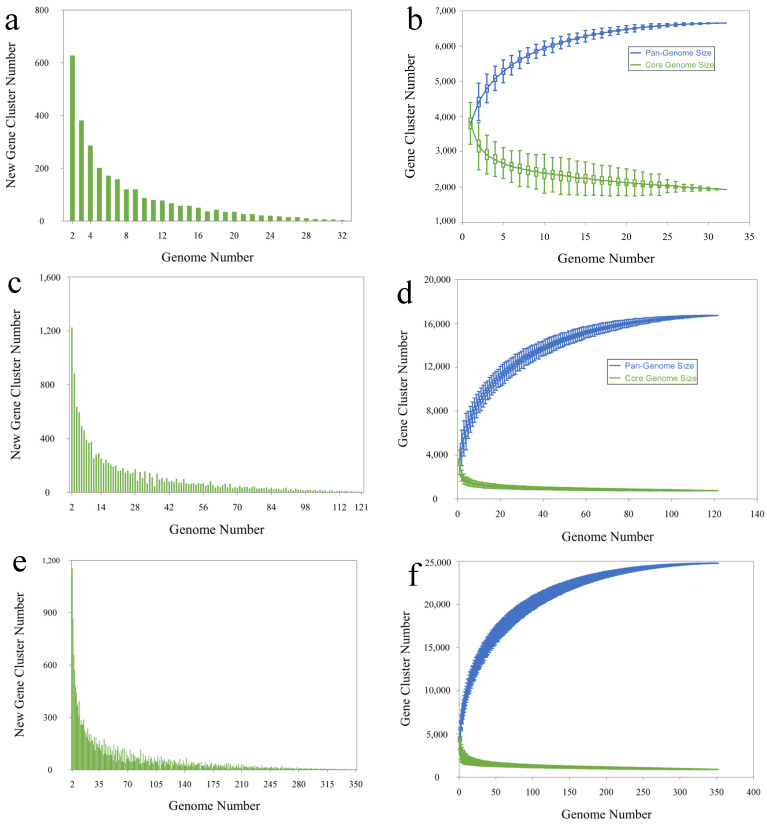
Pan-genome and core-genome dynamics of *Aeromonas*, *Bacillus*, and *Pseudomonas*. (**a**,**c**,**e**) Number of novel gene clusters detected per strain for each genus, showing pan-genome expansion patterns. (**b**,**d**,**f**) Accumulation curves for pan-genome (solid lines) and core-genome (dashed lines). *Pseudomonas* exhibits the highest pan-genome growth rate, reflecting high genomic plasticity, while *Aeromonas* shows the slowest expansion and a relatively stable core genome. *Bacillus* presents intermediate dynamics. These curves highlight the balance of core, accessory, and unique genes, illustrating genus-specific genome flexibility and potential as indicators of metal stress. Data derived from OrthoFinder and PanGP analysis.

The evolution of pan-genome and core genome sizes in *Aeromonas*, *Bacillus*, and *Pseudomonas* was characterized by dynamic patterns (Figure 4). All three genera exhibited the typical trend of pan-genome expansion, in which the number of novel gene clusters gradually decreased with increasing genome size, although expansion did not cease [15,16,47]. This pattern underscored their considerable genomic diversity and highlighted the ongoing acquisition of new genes during adaptive evolution.

Pan-Genome Comparison:

- The pan-genome of *Aeromonas* expanded at a relatively slow rate, with the fitted curve $y=834421x^{0.003}-830494$, and an R^2^ value of 0.987. The slowing increase in novel gene clusters indicated that although the pan-genome continued to grow, the rate of expansion was diminishing.
- In contrast, *Bacillus* exhibited a faster trajectory, with the fitted curve $y=79262x^{0.03}-7695$, and an R^2^ value of 0.992. Although the rate of increase also declined, the overall expansion remained more pronounced.
- *Pseudomonas* displayed the most rapid expansion, with the fitted curve $y=71284x^{0.04}-69862$, and an R^2^ value of 0.982. This indicated a highly open pan-genome, with new gene clusters continuously emerging as genome size increased. *Pseudomonas* displayed the highest growth rate among the three species.

In terms of pan-genome openness [48], *Pseudomonas* demonstrated a significantly higher growth rate than both *Aeromonas* and *Bacillus*, likely reflecting its broader ecological adaptability.

Core Genome Comparison:

- The core genome of *Aeromonas* followed an exponential decline, modeled by $y=1732e^{-0.17x}+2028$, and an R^2^ value of 0.951082. This suggested that as genome size increased, the number of core genes gradually decreased and ultimately stabilized.
- The core genome of *Bacillus* showed a more rapid decline, with the curve fitting the equation $y=2121e^{-0.16x}+867$, and an R^2^ value of 0.853. The number of core genes decreased more sharply and stabilized at a lower gene count.
- The core genome of *Pseudomonas* exhibited a slower decline, with the curve fitting the equation $y=1537e^{-0.02x}+1026$, and an R^2^ value of 0.953. In contrast to *Aeromonas* and *Bacillus*, *Pseudomonas* maintained a more stable core genome, with a less pronounced reduction in core gene numbers.

**Figure 5:**
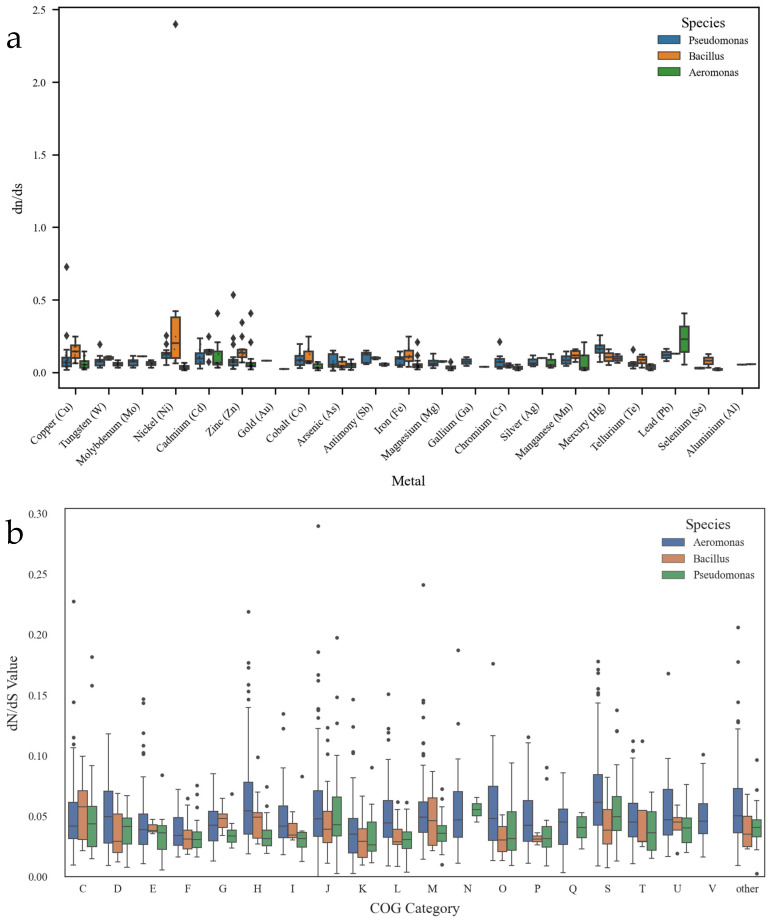
(**a**) dN/dS ratio of 23 metal resistance genes in *Aeromonas*, *Bacillus*, and *Pseudomonas*. (**b**) dN/dS ratio of core genes in *Aeromonas*, *Baciwllus*, and *Pseudomonas* based on COG classification.

Among the three, *Bacillus* exhibited the steepest decline, suggesting a smaller conserved functional gene set (Figure 4d), whereas *Aeromonas* and *Pseudomonas* retained a larger proportion of essential genes (Figure 4b,f). The relative proportions further supported this trend: 25% of the genes in *Aeromonas* were classified as core genes, compared with 4.6% in *Bacillus* and 4.3% in *Pseudomonas* (Figure S1b and Figure 5). The higher diversity observed in *Pseudomonas* likely reflected its larger genome size, while the reduced core gene set in *Bacillus*—derived from only 123 strains—highlighted its substantial genetic diversity [17]. The proportion of unique genes was also highest in *Bacillus* (22.9%), exceeding both *Aeromonas* and *Pseudomonas*, which may reflect extensive horizontal gene transfer events within this genus [49]. These contrasts further support the potential use of core/accessory ratios as bioindicators of selective pressures in contaminated environments.

Marked differences in homologous gene ratios were observed within *Aeromonas* species (Figure S2a). *A. veronii* and *A. hydrophila* exhibited high ratios, consistent with strong genomic similarity, likely attributable to their comparable ecological niches and lifestyles [15]. This genomic similarity likely extends to conserved heavy metal resistance genes shared between *A. veronii* and *A. hydrophila* [50]. In contrast, the ratio between *A. salmonicida* and *A. jandaei* was lower, suggesting that these species had experienced distinct selective pressures, resulting in substantial divergence. Within the genus *Bacillus* (Figure S2b), *B. cereus* and *B. thuringiensis* displayed relatively high homologous ratios, consistent with their close relationship as members of the *B. cereus* complex [51]. Conversely, *B. subtilis* and *B. licheniformis* exhibited lower ratios, reflecting pronounced genomic divergence associated with distinct ecological strategies. Their high similarity may reflect conserved metal detoxification strategies within the *B. cereus* complex [43]. For *Pseudomonas* (Figure S2c), homologous gene ratios were generally low, underscoring the considerable genomic diversity within the genus. Nevertheless, *P. aeruginosa* and *P. fluorescens* exhibited relatively high similarity, suggesting shared metabolic functions and ecological adaptability. By contrast, *P. putida* and *P. syringae* displayed lower ratios, highlighting substantial genomic divergence.

### 3.4. Functional Characterization of the Pan-Genome

The distribution of genes across functional categories revealed that the accessory genome contributed disproportionately to several categories, including transcription (K), amino acid transport and metabolism (E), carbohydrate transport and metabolism (G), secondary metabolite biosynthesis, transport, and metabolism (Q), cell wall/membrane/outer membrane biogenesis and modification (M), signal transduction mechanisms (T), and defense mechanisms (V) (Figure S3). In these categories, the number of genes in the accessory genome was generally much higher than in the core genome or unique genes, with particularly pronounced differences observed in *Pseudomonas* and *Bacillus* (Figure S3b,c). In the K category, all three genera exhibited significantly more genes in the accessory genome compared to the core genome (*p* < 0.01), indicating that transcriptional regulation may enable physiological adaptation under varying environmental conditions. Similarly, in categories E and G, especially in *Bacillus* and *Pseudomonas* (Figure S3b,c), accessory genome genes significantly outnumbered those in the core genome (*p* < 0.001 and *p* < 0.01, respectively). These results suggest that these genera possess greater diversity in nutrient acquisition and metabolic pathways, enhancing their adaptability to environments with fluctuating nutrient availability. In the Q category, *Pseudomonas* showed a markedly higher number of accessory genome genes relative to the core genome (*p* < 0.001), implying that its diversity of secondary metabolites may provide a competitive advantage for survival across diverse ecological niches. Likewise, *Pseudomonas* also harbored significantly more accessory genome genes in categories T and V (*p* < 0.01 and *p* < 0.001, respectively). This enrichment has been associated with heavy metal resistance, as T and V often include metal-sensing regulators and efflux pumps encoded in the accessory genome [52]. The enrichment of these categories indicates that *Pseudomonas* is well equipped to sense and rapidly respond to external environmental cues, while simultaneously maintaining strong resistance capacities, including tolerance to antibiotics and heavy metals. In category M (Figure S3a), both *Aeromonas* and *Pseudomonas* displayed significantly higher numbers of accessory genome genes compared with the core genome (*p* < 0.01). This finding suggests that these genera exhibit diversity in cell wall and membrane modification, enabling them to adapt more effectively to complex and variable environments. Such structural flexibility is known to enhance bacterial tolerance to toxic metal ions by modifying membrane permeability and reducing intracellular accumulation [53]. Overall, these results align with previous studies emphasizing the pivotal role of the accessory genome in facilitating rapid adaptation to fluctuating environmental pressures by enhancing metabolic versatility, antibiotic resistance, and cellular structural plasticity [15,54]. This is particularly relevant for metal-contaminated environments, where accessory gene pools frequently encode horizontally acquired metal resistance operons [55]. Taken together, this evidence supports the view that the accessory genome constitutes a critical factor in the ecological success and evolutionary fitness of bacterial species in diverse habitats [19].

### 3.5. Evolution of Core and Metal Resistance Genomes

The distributions of dN/dS values for heavy metal resistance genes in *Aeromonas*, *Bacillus*, and *Pseudomonas* revealed distinct patterns (Figure 5a). Overall, most metal resistance genes exhibited dN/dS values below 1, indicating that they were predominantly subjected to purifying selection during evolution, thereby preserving their essential functions. For certain metals, including Cd, Pb, and Zn, *Bacillus* displayed relatively high dN/dS values, suggesting that these genes may have experienced positive selection, enhancing the ability of the genus to cope with heavy metal-induced stress [56]. Likewise, *Pseudomonas* exhibited elevated dN/dS values for some resistance genes, such as those associated with iron (Fe) and magnesium (Mg), possibly reflecting adaptive responses to environmental pressures. Such diversification of resistance genes may have strengthened its survival in complex and variable environments. Collectively, these findings suggest that both *Bacillus* and *Pseudomonas* have likely evolved positive selection mechanisms to optimize metal resistance, thereby enhancing their adaptability and ecological fitness in metal-contaminated environments [57]. Although core heavy metal resistance (HMR) genes in *Pseudomonas* are largely conserved under purifying selection (dN/dS < 1), the genus exhibits high genomic plasticity via dynamic expansion of accessory gene families, gene acquisition/loss, and diverse secondary metabolite clusters. This allows functional adaptation to varying metal stresses without altering the sequences of core resistance genes, reconciling apparent sequence stability with a fluid pan-genome.

The distribution of dN/dS values across different COG functional categories in the three genera is presented in Figure 5b. In general, most categories displayed dN/dS values below 1, indicating that the majority of functional genes have been maintained by purifying selection to preserve basic physiological processes [15]. However, several categories exhibited signs of adaptive evolution. In category Q, *Pseudomonas* showed significantly higher dN/dS values compared to the other genera, suggesting that secondary metabolite biosynthesis-related genes may have been under stronger adaptive selection, thereby aiding its survival in dynamic and complex environments [58]. In category E, *Bacillus* displayed relatively high dN/dS values, implying that its metabolism-related genes may have undergone positive selection, facilitating adaptation to environments with fluctuating nutrient availability [59]. By contrast, *Aeromonas* exhibited higher dN/dS values in category V, suggesting that its defense-related genes likely experienced strong positive selection, thereby enhancing resilience to environmental stress [15].

### 3.6. Gene Gain and Loss for the Metal Resistance Genomes and Pan-Genome

Overall, *Pseudomonas* exhibited a markedly higher degree of gene family expansion compared with the other two genera, with particularly pronounced increases in specific categories (Figure S4a). Notably, expansions in COG functional categories Q and V were most significant. The amplification of secondary metabolite–related genes in category Q suggests that *Pseudomonas* may harbor more complex metabolic functions [60], enabling the synthesis of diverse secondary metabolites—including antibiotics, toxins, and metal chelates—that confer a competitive advantage over other microorganisms. Likewise, expansion in category V reflects enhanced survival under environmental stress, such as heavy metal contamination and antibiotic exposure, through the enrichment of defense-related genes. This trend may also be linked to the induction of distinct gene sets under varying heavy metal and antibiotic conditions, enabling *Pseudomonas aeruginosa* to transition into phenotypes that are resistant to both stressors while exhibiting reduced virulence, such as in mucoid biofilm formation [61]. Collectively, these findings indicate that *Pseudomonas* has undergone large-scale amplification of functional gene families, underpinning its adaptability, resistance mechanisms, and ecological competitiveness [54]. The expansion of these families likely enhances both ecological diversity and survival capacity in complex environments. Metagenomic studies have similarly reported co-enrichment (MRG) with secondary-metabolite or metallophore biosynthetic clusters under metal stress [62].

In contrast, *Bacillus* and *Aeromonas* exhibited a more balanced pattern of gene family expansion and contraction, suggesting limited large-scale amplification. This stability may be associated with more conservative ecological strategies, particularly in *Aeromonas*, which relied heavily on a stable core genome to maintain physiological functions while showing fewer family-level changes [15]. This stability facilitated survival in homogeneous environments, consistent with lower dN/dS ratios, indicating reduced substitution rates and limited genomic diversity [63]. With only 32 *Aeromonas* strains currently identified, the core genome comprised approximately 25% of total genes, suggesting a relatively stable gene repertoire. By contrast, *Bacillus* exhibited moderate expansion in specific metabolism-related families, especially in categories E and G, reflecting enhanced metabolic flexibility that may facilitate adaptation to environments with variable nutrient availability. Previous studies have similarly reported increases in orthologous clusters related to carbohydrate transport and metabolism in *Bacillus* [64]. Consistently, field surveys have shown that *Aeromonas* and *Bacillus* occurred in metal-impacted waters and soils with relatively stable resistance patterns detectable by metagenomics, albeit typically with a narrower breadth than *Pseudomonas* [65].

Comparative phylogenetic analyses revealed marked differences in gene family dynamics and metal resistance gene distribution across the three genera (Figure S4b–d). In *Aeromonas*, gene family expansion and contraction were generally balanced, indicating reliance on stable core functions, although moderate expansion occurred in heavy metal resistance genes. By contrast, *Bacillus* showed more frequent expansion than contraction, particularly in certain phylogenetic branches, suggesting that increased metabolic flexibility and stress tolerance were achieved through gene family expansion. Heatmap analyses further indicated that some *Bacillus* strains contained elevated numbers of iron- and zinc-resistance genes, underscoring their adaptability to element-rich environments. In *Pseudomonas*, however, gene family contraction predominated over expansion. These contracted families likely represented functions maladaptive under specific ecological conditions, leading to their elimination [66]. Nevertheless, *Pseudomonas* exhibited a significant increase in resistance genes for Cu and nickel (Ni), suggesting selective retention of traits crucial for survival under heavy metal stress [67]. These lineage-resolved patterns align with observations that *Pseudomonas* proliferates in metal-contaminated sediments and soils; correspondingly, metagenomic datasets from polluted sites have often shown elevated *Pseudomonas*-linked MRGs relative to co-occurring taxa. Such concordance supports using taxon-specific MRG profiles as quantitative readouts of local metal pressure [68].

Within individual species, considerable strain-level variation was also evident. In *Aeromonas*, *A. veronii* displayed expansion in metabolism-related families, likely reflecting adaptation to diverse aquatic habitats [15]. By contrast, *A. hydrophila* showed pronounced expansion in Cd- and Pb-resistance genes [69,70], consistent with enhanced survival in metal-contaminated waters. Among *Bacillus* strains, *B. cereus* exhibited expansion in carbohydrate metabolism families, suggesting adaptation to nutrient-rich conditions, while *B. subtilis* expanded Fe- and Mn-resistance genes, reflecting enhanced tolerance to metal-enriched environments [71]. Within *Pseudomonas*, *P. aeruginosa* primarily exhibited contraction of metabolism-related families, whereas *P. fluorescens* and *P. putida* demonstrated significant expansion in Cu- and Ni-resistance genes [13], underscoring their high adaptability to industrial pollution and other metal-enriched habitats. Metagenomic correlations between local metal spectra and specific MRGs (e.g., *czcA*, *copA*) have been documented, indicating that strain-level resistance modules can mirror site-specific metal availability and bioavailability [62].

## 4. Conclusions

Comparative pangenomic analysis provides insights into genome openness, resistance gene diversity, and evolutionary constraints across microbial taxa. Among the studied genera, Cu and Zn resistance genes were the most abundant. All three genera exhibited open pangenomes, with *Pseudomonas* having the highest accessory genome proportion and *Aeromonas* the highest core gene retention. Most resistance genes were under purifying selection (dN/dS < 1), though adaptive signals appeared in Cd-, Zn-, and Fe-related genes in *Pseudomonas* and *Bacillus*. Gene family expansion was most pronounced in *Pseudomonas*, especially for Cu/Ni resistance and NRP biosynthesis clusters, while *Bacillus* showed moderate metabolic family expansion, and *Aeromonas* remained genomically stable. Collectively, our analyses delineate clear genomic baselines for *Aeromonas*, *Bacillus*, and *Pseudomonas*, linking their resistance gene repertoires, selective pressures, and gene family dynamics to metal-specific ecological contexts. These baselines reveal that Pseudomonas possesses the most plastic and metal-enriched genome, Bacillus adapts through moderate metabolic diversification, and *Aeromonas* maintains genomic stability as a core ecological generalist. These findings provide a quantitative framework for predicting microbial adaptation under heavy metal stress and offer reference datasets for metagenomic surveillance of metal-contaminated environments.

## Data Availability

Dataset available upon request from the authors.

## Supplementary Materials

The following supporting information can be downloaded at: https://www.mdpi.com/article/10.3390/biology15100751/s1, Figure S1: (a), (b), and (c) show the proportions of core genes, accessory genes, and unique genes in *Aeromonas*, *Bacillus*, and *Pseudomonas*, respectively; Figure S2: (a), (b), and (c) show the distribution of COG categories of core genes, accessory genes, and unique genes in *Aeromonas*, *Bacillus*, and *Pseudomonas*, respectively; Figure S3: (a), (b), and (c) show the percentage of homologous gene families for each pair of *Aeromonas*, *Bacillus*, and *Pseudomonas*; Figure S4: (a) COG category distribution of significantly expanded and contracted gene families in *Aeromonas*, *Bacillus*, and *Pseudomonas*. (b), (c), and (d) show the phylogenetic trees and statistics of expansion and contraction of metal resistance gene families in *Aeromonas*, *Bacillus*, and *Pseudomonas*, respectively.

## Abbreviations

MRG	Metal Resistance Genes
NRP	Non-ribosomal peptide
COG	Clusters of Orthologous Groups
