# Supplementary material for: Evolutionary Strategies for Heavy Metal Resistance: Genomic Plasticity in Pseudomonas Versus Stability in Aeromonas and Bacillus
Source: Biology (Basel). 2026 May 9;15(10):751. doi: 10.3390/biology15100751 (PMC13203726; doi:10.3390/biology15100751)
Supplement: Supplementary file 1 [file biology-15-00751-s001.zip › biology-4255380-supplementary.pdf]

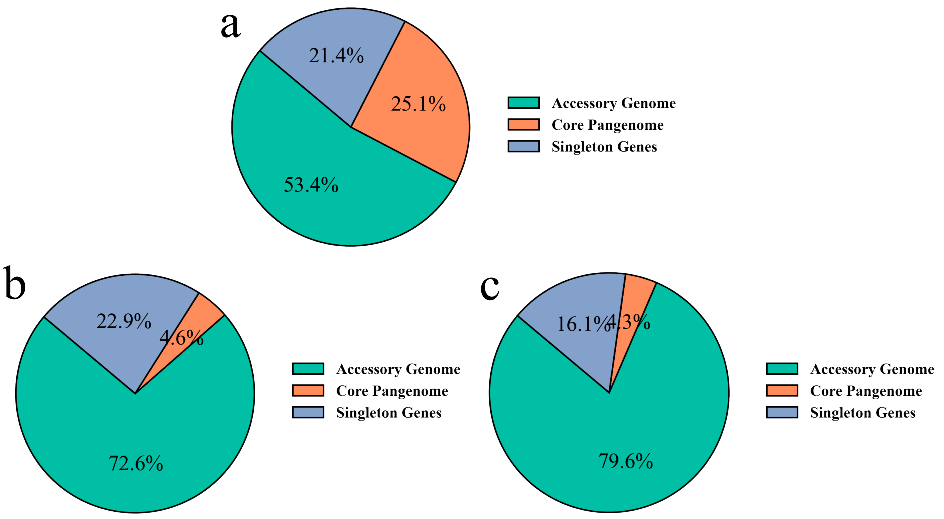

Figure.S1 (a), (b), and (c) show the proportions of core genes, accessory genes, and unique genes in *Aeromonas*, *Bacillus*, and *Pseudomonas*, respectively.

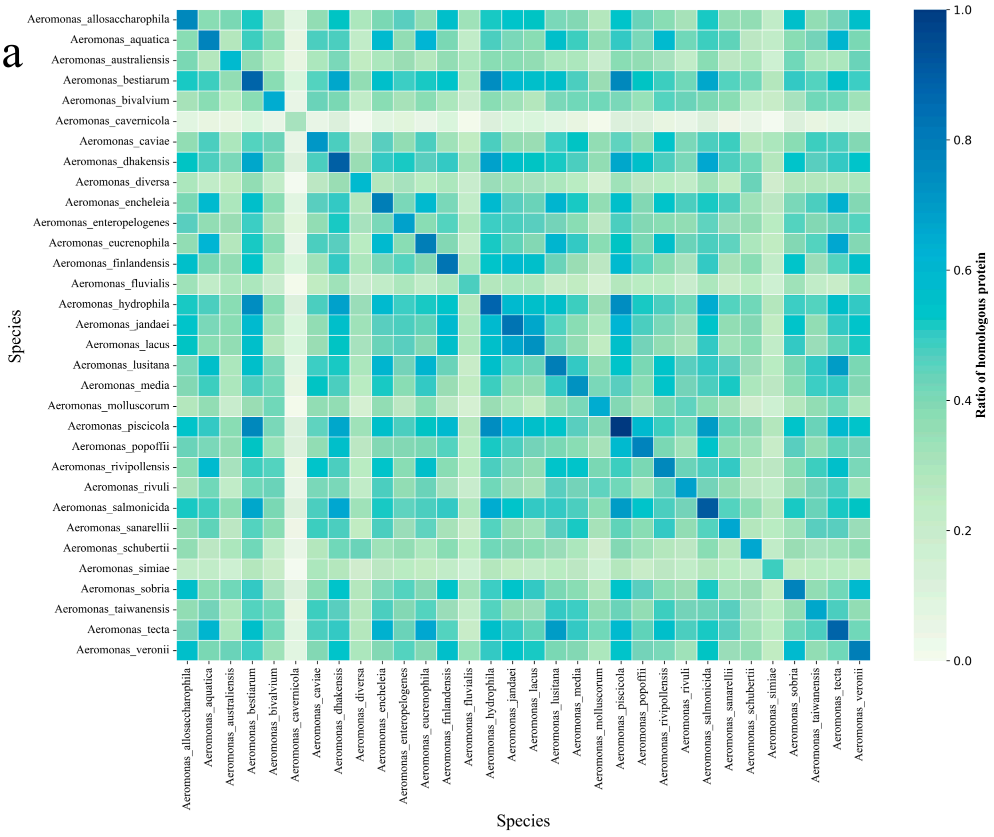

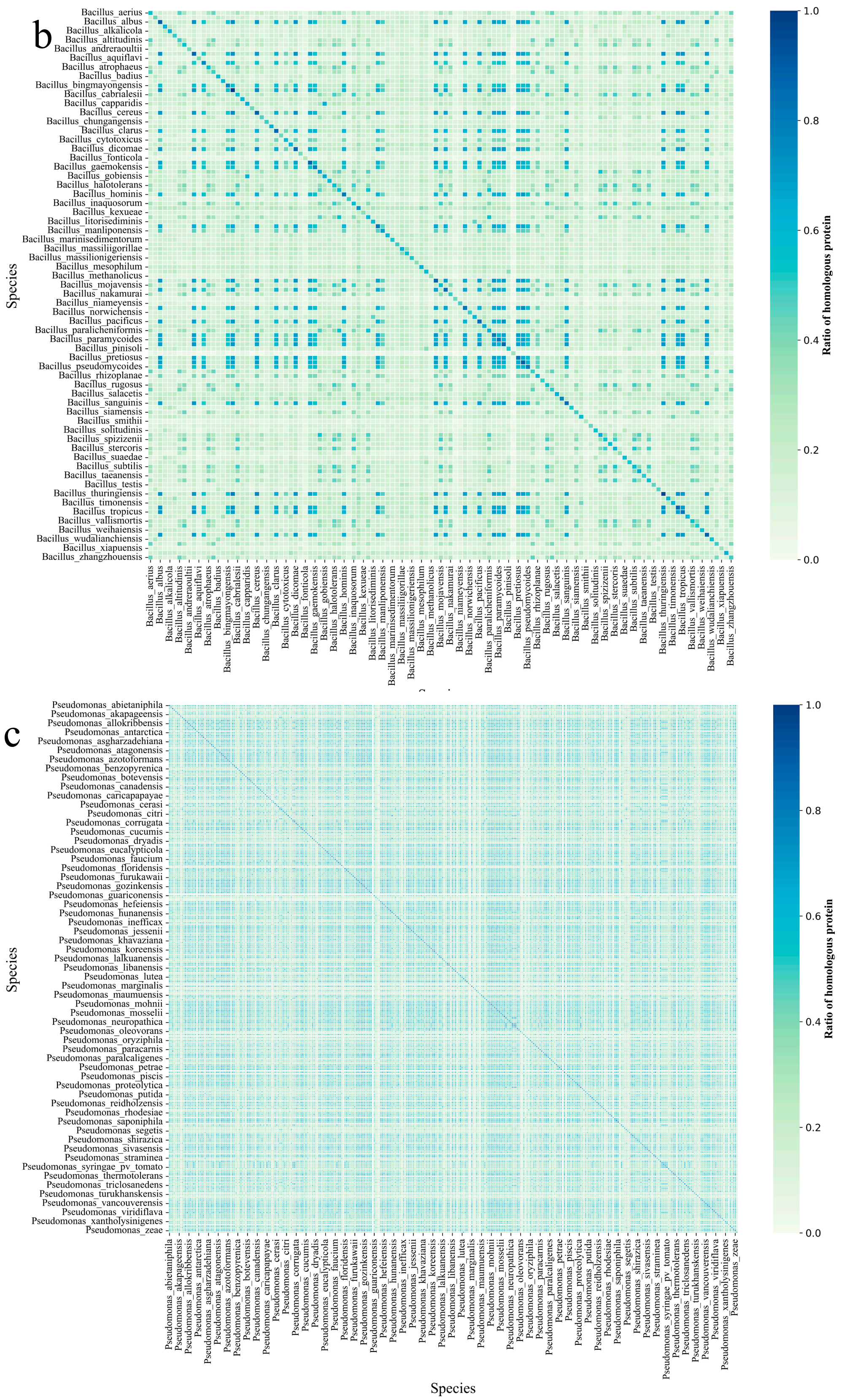

Figure.S2 (a), (b), and (c) show the distribution of COG categories of core genes, accessory genes, and unique genes in *Aeromonas*, *Bacillus*, and *Pseudomonas*, respectively.

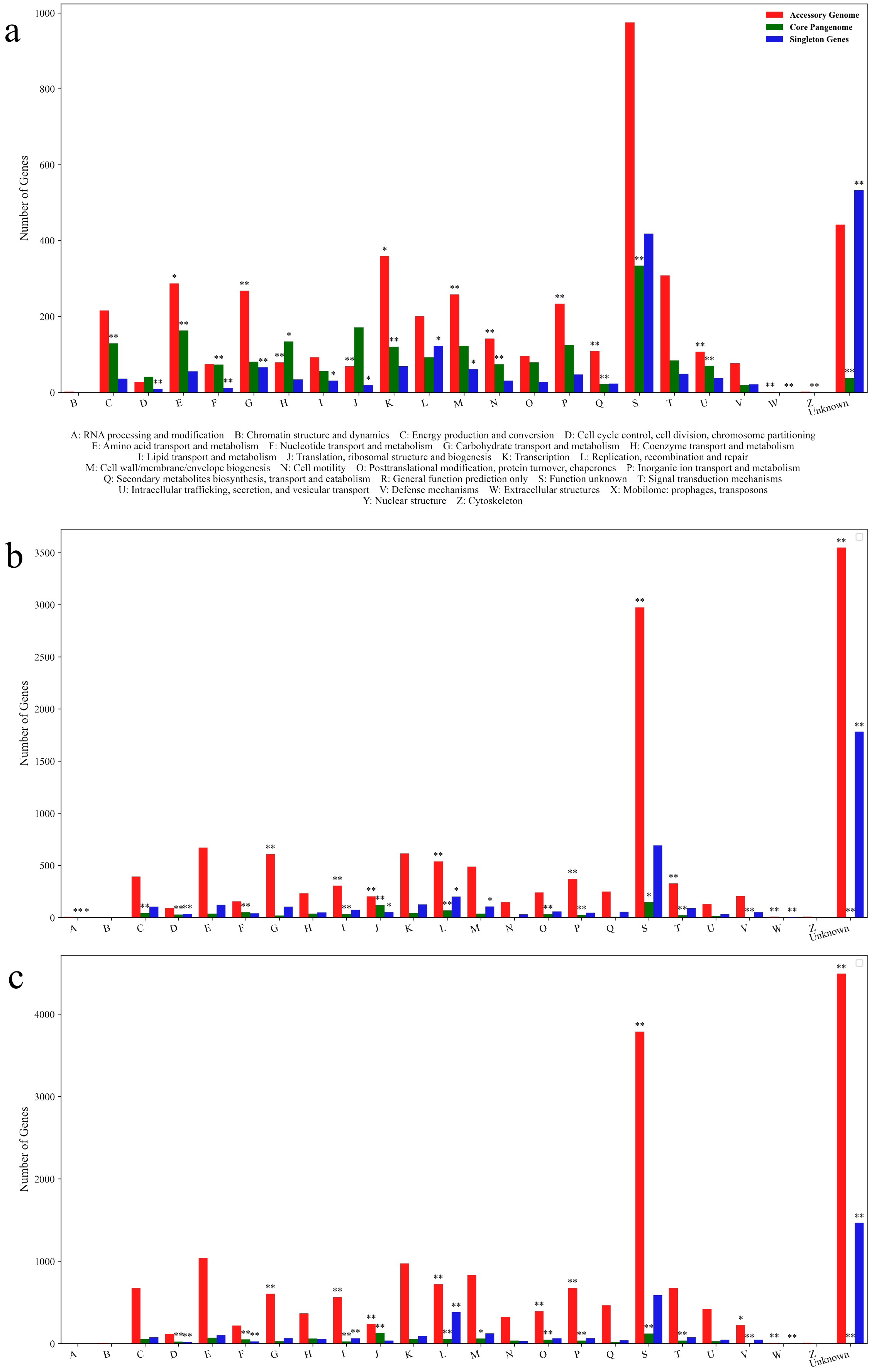
a
Accessory Genome
Core Pangenome
Singleton Genes
Number of Genes
Unknown
A: RNA processing and modification B: Chromatin structure and dynamics C: Energy production and conversion D: Cell cycle control, cell division, chromosome partitioning
E: Amino acid transport and metabolism F: Nucleotide transport and metabolism G: Carbohydrate transport and metabolism H: Coenzyme transport and metabolism
I: Lipid transport and metabolism J: Translation, ribosomal structure and biogenesis K: Transcription L: Replication, recombination and repair
M: Cell wall/membrane/envelope biogenesis N: Cell motility O: Posttranslational modification, protein turnover, chaperones P: Inorganic ion transport and metabolism
Q: Secondary metabolites biosynthesis, transport and catabolism R: General function prediction only S: Function unknown T: Signal transduction mechanisms
U: Intracellular trafficking, secretion, and vesicular transport V: Defense mechanisms W: Extracellular structures X: Mobilome: prophages, transposons
Y: Nuclear structure Z: Cytoskeleton
b
Number of Genes
Unknown
c
Number of Genes
Unknown

Figure.S3 (a), (b), and (c) show the percentage of homologous gene families for each pair of *Aeromonas*, *Bacillus*, and *Pseudomonas*.

a

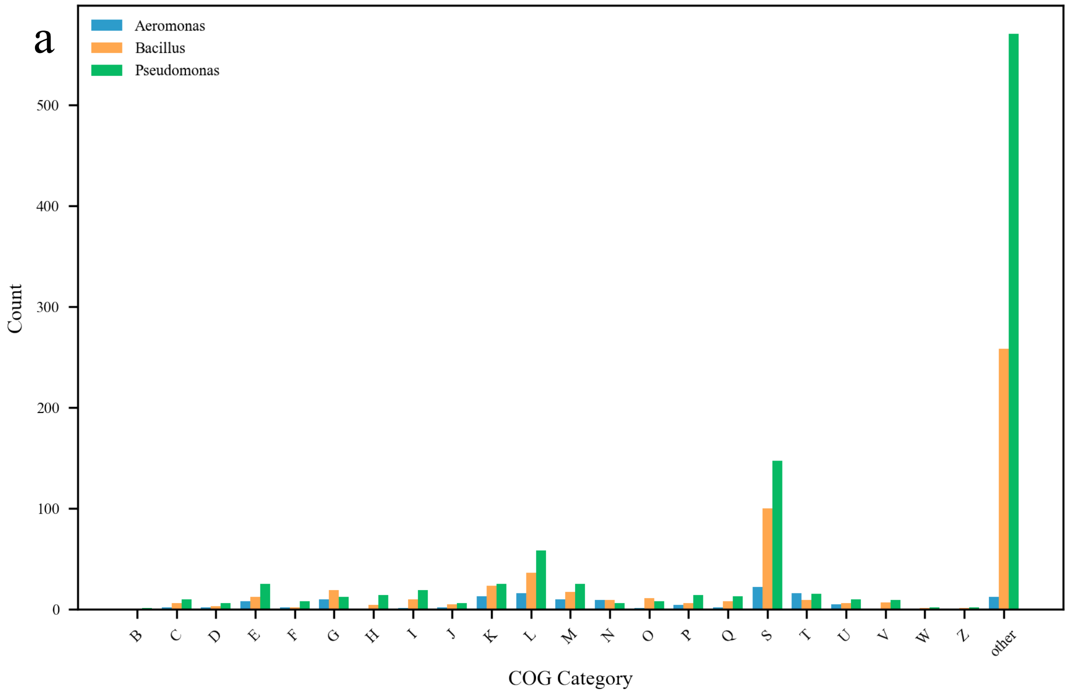

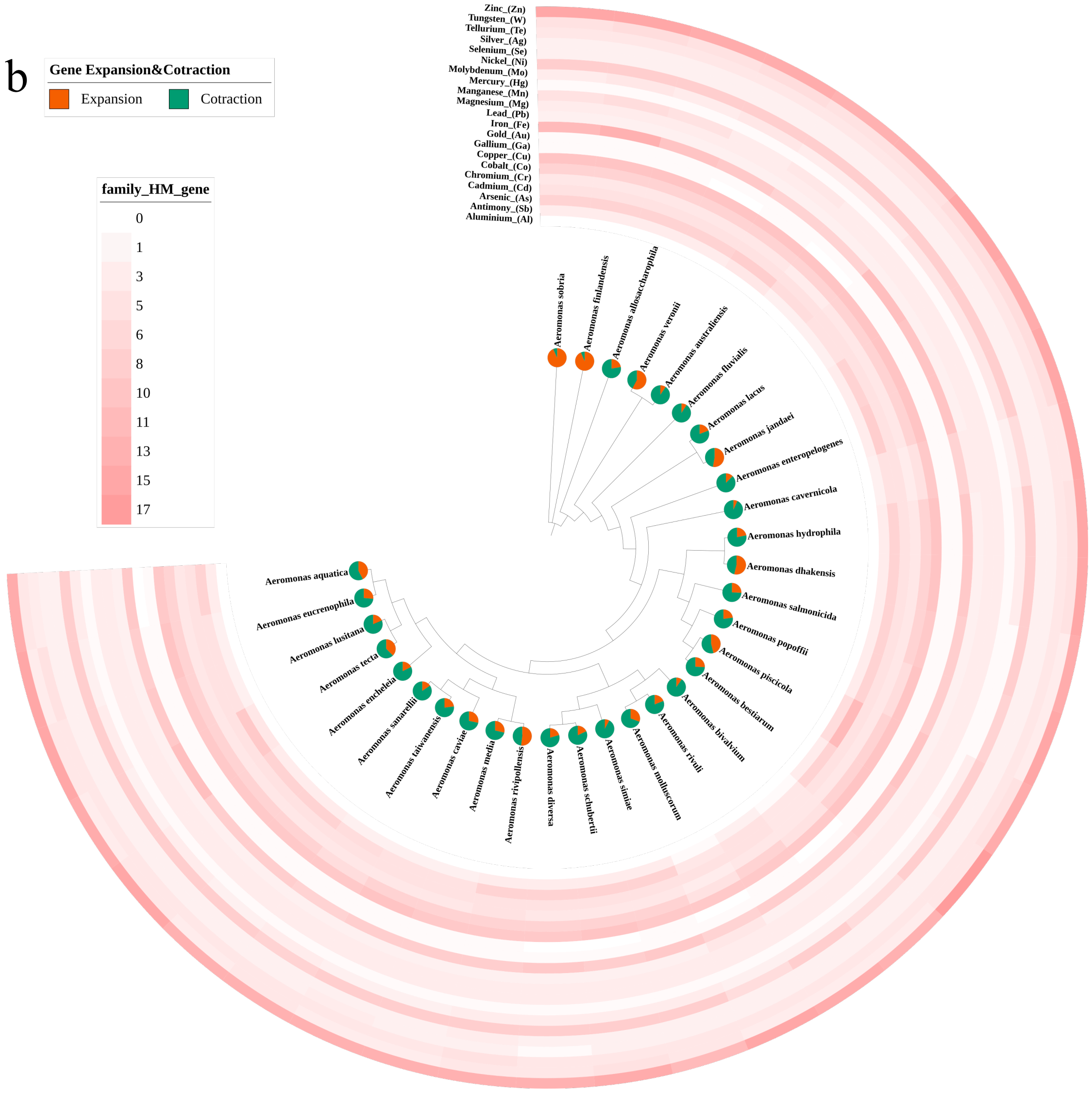
b
Gene Expansion&Cotraction
Expansion
Cotraction
family_HM_gene
0
1
3
5
6
8
10
11
13
15
17
Zinc_(Zn)
Tungsten_(W)
Tellurium_(Te)
Silver_(Ag)
Selenium_(Se)
Nickel_(Ni)
Molybdenum_(Mo)
Mercury_(Hg)
Manganese_(Mn)
Magnesium_(Mg)
Lead_(Pb)
Iron_(Fe)
Gold_(Au)
Gallium_(Ga)
Copper_(Cu)
Cobalt_(Co)
Chromium_(Cr)
Cadmium_(Cd)
Arsenic_(As)
Antimony_(Sb)
Aluminium_(Al)
Aeromonas sobria
Aeromonas finlandensis
Aeromonas allosaccharophila
Aeromonas veronii
Aeromonas australiensis
Aeromonas fluvialis
Aeromonas lacus
Aeromonas jandaei
Aeromonas enteropelogenes
Aeromonas cavernicola
Aeromonas hydrophila
Aeromonas dhakensis
Aeromonas salmonicida
Aeromonas popoffii
Aeromonas piscicola
Aeromonas bestiarum
Aeromonas bivalvium
Aeromonas rivuli
Aeromonas molluscorum
Aeromonas simiae
Aeromonas schubertii
Aeromonas diversa
Aeromonas rivipollensis
Aeromonas media
Aeromonas caviae
Aeromonas taiwanensis
Aeromonas sanarellii
Aeromonas encheleia
Aeromonas tecta
Aeromonas lusitana
Aeromonas eucrenophila
Aeromonas aquatica

c

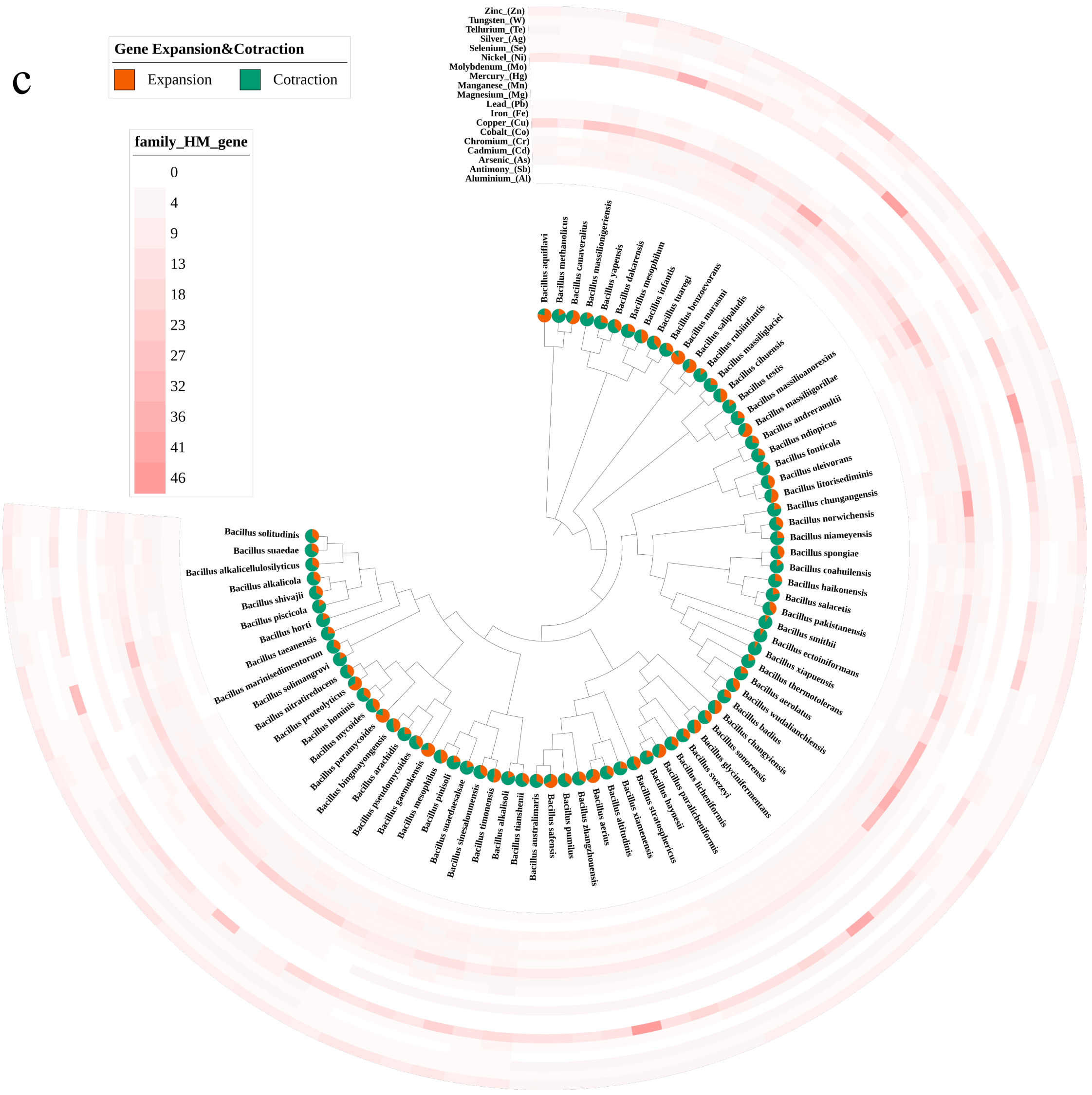

Gene Expansion&Cotraction
Expansion
Cotraction
family_HM_gene
0
4
9
13
18
23
27
32
36
41
46
Zinc_(Zn)
Tungsten_(W)
Tellurium_(Te)
Silver_(Ag)
Selenium_(Se)
Nickel_(Ni)
Molybdenum_(Mo)
Mercury_(Hg)
Manganese_(Mn)
Magnesium_(Mg)
Lead_(Pb)
Iron_(Fe)
Copper_(Cu)
Cobalt_(Co)
Chromium_(Cr)
Cadmium_(Cd)
Arsenic_(As)
Antimony_(Sb)
Aluminium_(Al)
Bacillus aquiflavi
Bacillus methanolicus
Bacillus canaveralius
Bacillus massilionigeriensis
Bacillus yapensis
Bacillus dakarensis
Bacillus mesophilum
Bacillus infantis
Bacillus tuaregi
Bacillus benzoevorans
Bacillus marasmi
Bacillus salipaludis
Bacillus rubiinfantis
Bacillus massiliglaciei
Bacillus chuensis
Bacillus testis
Bacillus massilioanorexius
Bacillus massiliigorillae
Bacillus andreraoultii
Bacillus ndiopicus
Bacillus fonticola
Bacillus oleivorans
Bacillus litorisediminis
Bacillus chungangensis
Bacillus norwichensis
Bacillus niameyensis
Bacillus spongiae
Bacillus coahuilensis
Bacillus haikouensis
Bacillus salacetis
Bacillus pakistanensis
Bacillus smithii
Bacillus ectoiniformans
Bacillus xiapuensis
Bacillus thermotolerans
Bacillus aerolatus
Bacillus wudalianchiensis
Bacillus badius
Bacillus changyiensis
Bacillus sonorensis
Bacillus glycinifermentans
Bacillus swezeyi
Bacillus licheniformis
Bacillus paralicheniformis
Bacillus haynesii
Bacillus stratosphericus
Bacillus xiamenensis
Bacillus altitudinis
Bacillus aerius
Bacillus zhangzhouensis
Bacillus pumilus
Bacillus safensis
Bacillus australimaris
Bacillus tianshenii
Bacillus alkalisoli
Bacillus timonensis
Bacillus sinesaloumensis
Bacillus suaedaesalsae
Bacillus pinisoli
Bacillus mesophilus
Bacillus gaemokensis
Bacillus pseudomycoides
Bacillus arachidis
Bacillus bingmayongensis
Bacillus paramycoides
Bacillus mycoides
Bacillus hominis
Bacillus proteolyticus
Bacillus nitratireducens
Bacillus solimangrovi
Bacillus marinisedimentorum
Bacillus taeanensis
Bacillus horti
Bacillus piscicola
Bacillus shivajii
Bacillus alkalicola
Bacillus alkalicellulosilyticus
Bacillus suaedae
Bacillus solitudinis

d

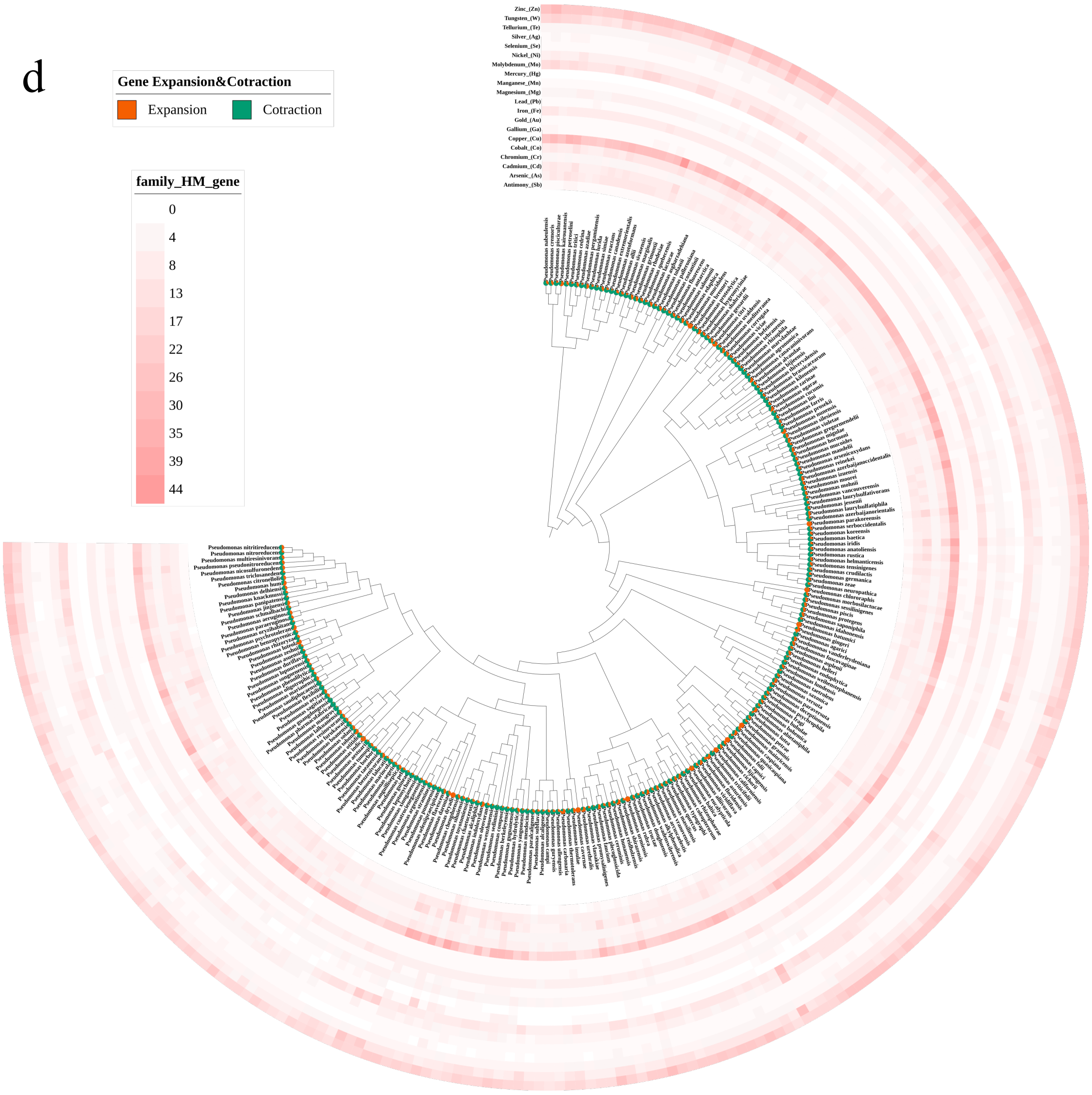

Figure.S4 (a) COG category distribution of significantly expanded and contracted gene families in *Aeromonas*, *Bacillus*, and *Pseudomonas*. (b), (c), and (d) show the phylogenetic trees and statistics of expansion and contraction of metal resistance gene families in *Aeromonas*, *Bacillus*, and *Pseudomonas*, respectively.
